# High bone marrow *ID2* expression predicts poor chemotherapy response and prognosis in acute myeloid leukemia

**DOI:** 10.18632/oncotarget.20559

**Published:** 2017-09-01

**Authors:** Jing-Dong Zhou, Ji-Chun Ma, Ting-Juan Zhang, Xi-Xi Li, Wei Zhang, De-Hong Wu, Xiang-Mei Wen, Zi-Jun Xu, Jiang Lin, Jun Qian

**Affiliations:** ^1^ Department of Hematology, Affiliated People’s Hospital of Jiangsu University, Zhenjiang, Jiangsu, People’s Republic of China; ^2^ Laboratory Center, Affiliated People’s Hospital of Jiangsu University, Zhenjiang, Jiangsu, People’s Republic of China; ^3^ The Key Lab of Precision Diagnosis and Treatment of Zhenjiang City, Zhenjiang, Jiangsu, People’s Republic of China; ^4^ Department of Hematology, The Third People’s Hospital of KunShan City, Suzhou, Jiangsu, People’s Republic of China

**Keywords:** ID2, expression, prognosis, TCGA, AML

## Abstract

Dysregulation of ID proteins is a frequent event in various human cancers and has a direct role in cancer initiation, maintenance, progression and drug resistance. Our previous study has revealed *ID1* expression and its prognostic value in acute myeloid leukemia (AML). Herein, we further reported *ID2* expression and its clinical significance in AML. Real-time quantitative PCR was performed to detect *ID2* transcript level in bone marrow mononuclear cells of 145 *de novo* AML patients. *ID2* expression was significantly up-regulated in AML patients compared with controls. *ID2* overexpression occurred with the highest frequency in poor karyotype (10/17, 59%), lower in intermediate karyotype (35/83, 42%), and the lowest in favorable karyotype (7/40, 18%). Moreover, high *ID2* expression correlated with lower complete remission (CR) rate, shorter overall survival, and acted as an independent prognostic biomarker in whole-cohort AML and non-M3-AML patients. Importantly, the prognostic value of *ID2* expression in AML was validated by The Cancer Genome Atlas (TCGA) data. In the follow-up of patients, *ID2* expression at CR phase was decreased than at the time of diagnosis, and was increased again at the time of relapse. These findings demonstrated that bone marrow *ID2* overexpression was a frequent event in AML patients, and predicts poor chemotherapy response and prognosis.

## INTRODUCTION

As one of the most common type of hematological malignancies, acute myeloid leukemia (AML) is clinically, cytogenetically, and molecularly heterogeneous disease [1]. Despite intensive treatments with chemotherapy drugs and/or hematopoietic stem cell transplantation, less than half of AML patients are long time survivors [1, 2]. Cytogenetic abnormalities play vital roles in the pathogenesis of AML, and also provide powerful prognostic information [3]. Recently, molecular biological alterations including gene mutations and abnormal gene expression have been found as potential prognostic biomarkers in AML [4]. Therefore, identification of novel biomarkers could give us better understanding of the process of leukemogenesis, and are helpful to design effective therapeutic strategies for the patients with AML.

Inhibitor of DNA binding (ID) proteins are functional inhibitors of the basic helix-loop-helix, ETS and paired box transcription factors and thereby inhibit the transcription of lineage-specific and cell cycle-inhibitory genes that control the timing of cell fate determination and differentiation in stem and progenitor cells during normal development [5]. Dysregulation of ID proteins is frequent events in various human cancers and has a direct role in cancer initiation, maintenance, progression and drug resistance [5]. *ID* gene family is composed of four members (*ID1*, *ID2*, *ID3* and *ID4*), and each member plays a different role in carcinogenesis [5]. Our previous study showed that *ID1* overexpression was associated with higher risk karyotype classification and was a prognostically adverse indicator in young AML excluded M3 subtypes (non-M3-AML) patients [6]. In addition, *ID4* acted as a tumor suppressor in AML and epigenetic dysregulation of *ID4* independently affected clinical outcome in patients with AML [7].

Herein, we explored *ID2* expression and its clinical significance in AML patients. Overexpression of *ID2* was a frequent event in AML patients, and was associated with higher karyotype risks. Moreover, high bone marrow (BM) *ID2* expression predicts poor chemotherapy response and prognosis in AML patients.

## RESULTS

### ID2 expression in AML

**Figure 1:**
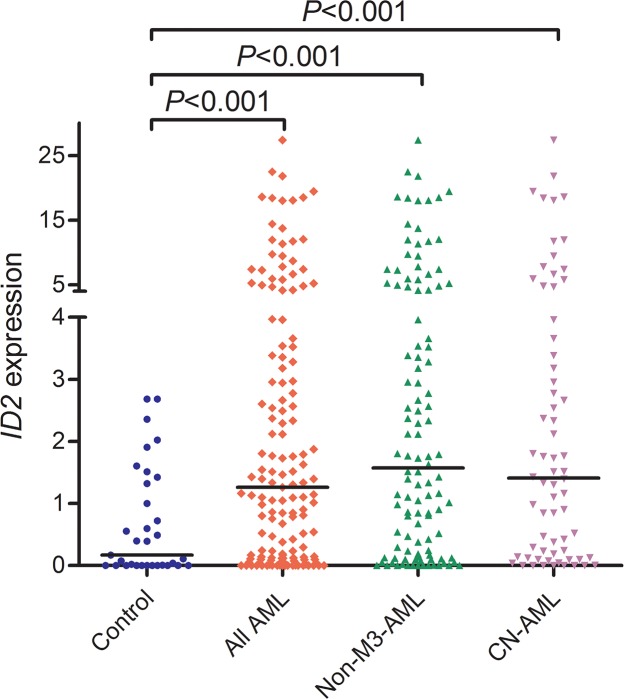
*ID2* expression in controls, whole-cohort AML, non-M3-AML, and CN-AML patients

From real-time quantitative PCR (RQ-PCR) analysis, *ID2* mRNA level in AML patients (median: 1.259, range: 0.000-27.387) was significantly higher than controls (median: 0.171, range: 0.000-2.684), which showed that *ID2* expression was significantly up-regulated in all AML (*P*<0.001, Figure 1). Additionally, up-regulation of *ID2* expression was also observed in non-M3-AML and cytogenetically normal AML (CN-AML) (both *P*<0.001, Figure 1). Moreover, *ID2* expression was positively associated with *ID1* (R=0.420, *P*<0.001, n=145) and *ID4* (R=0.245, *P*=0.001, n=145) expression in AML patients.

### Discriminative capacity of ID2 expression

**Figure 2:**
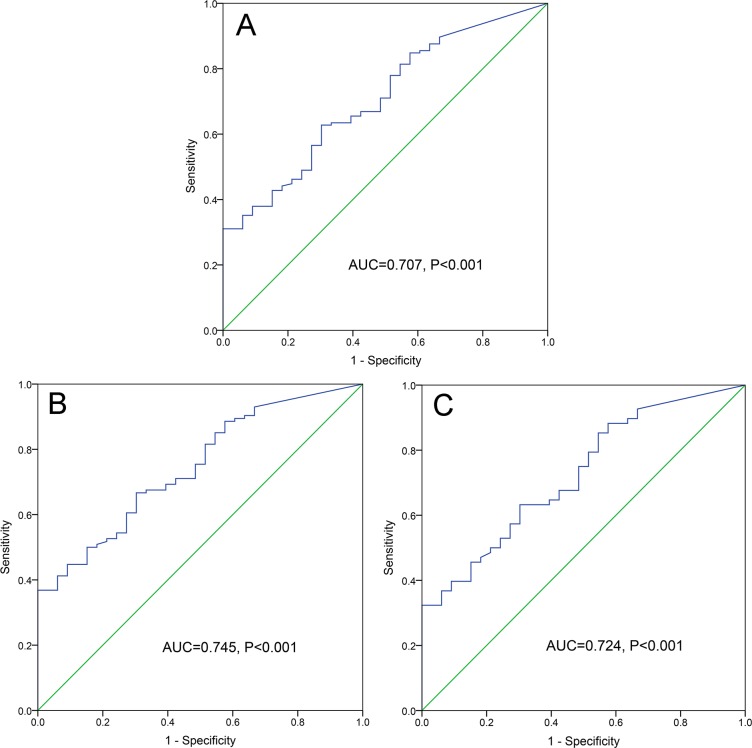
ROC curve analysis of *ID2* expression for discriminating AML patients form controls **(A)** whole-cohort AML patients; **(B)** non-M3-AML patients. **(C)** CN-AML patients.

By receiver operating characteristic curve (ROC) analysis, *ID2* expression with an area under the ROC curve (AUC) value of 0.707 [95% confidence interval (CI): 0.617-0.797] might serve as a potential biomarker for distinguishing AML form controls (*P*<0.001, Figure 2A). Moreover, the discriminative capacity of *ID2* expression was also observed among non-M3-AML (AUC=0.745, 95% CI: 0.658-0.832, *P*<0.001, Figure 2B) and CN-AML (AUC=0.724, 95% CI: 0.623-0.825, *P*<0.001, Figure 2C) patients.

### Correlation of ID2 expression with clinical characteristics

In order to analyze clinical relevance of *ID2* expression in AML, the whole-cohort AML patients were divided into two groups (*ID2*^high^ and *ID2*^low^) by the cut-off value (defined due to the sensitivity+specificity-1 was the highest value) based on ROC analysis. The comparison of clinical manifestations and laboratory features between *ID2*^high^ and *ID2*^low^ groups of AML patients was presented in Table 1. *ID2*^high^ patients had significantly older age (*P*=0.001) and a tendency of higher white blood cells (*P*=0.068). Significant differences were also observed in the distributions of French-American-British (FAB) and World Health Organization (WHO) classifications (*P*=0.009 and 0.016, respectively). High *ID2* expression occurred with the lowest frequency in M3 or t(15;17) subtype (4/31, 13%), which was lower than M0/M1/M2 subtypes (25/64, 39%) (*P*=0.010) and M0/M1/M2/M4/M5/M6 subtypes (51/114, 45%) (*P*=0.001). Moreover, among the karyotype classifications, *ID2*^high^ patients occurred with the highest frequency in poor karyotype (10/17, 59%), lower in intermediate karyotype (35/83, 42%), and the lowest in favorable karyotype (7/40, 18%) (*P*=0.004).

**Table 1:** Comparison of clinical manifestations and laboratory features between the AML patients with low and high *ID2* expression

Patient’s parameters	*ID2*^low^ (n=90)	*ID2*^high^ (n=55)	*P* value
Sex, male/female	51/39	33/22	0.731
Median age, years (range)	51 (18-87)	65 (18-93)	0.001
Median WBC, ×10^9^/L (range)	7.5 (0.3-528.0)	20.7 (1.1-197.7)	0.068
Median hemoglobin, g/L (range)	75 (34-138)	75 (32-142)	0.349
Median platelets, ×10^9^/L (range)	39 (3-264)	50 (4-447)	0.195
Median BM blasts, % (range)	43.5 (1.0-109.0)	47.8 (6.0-94.5)	0.489
FAB classifications			0.009
M0	1 (1%)	0 (0%)	
M1	7 (8%)	3 (5%)	
M2	31 (34%)	22 (40%)	
M3	27 (30%)	4 (7%)	
M4	14 (16%)	17 (31%)	
M5	7 (8%)	8 (15%)	
M6	3 (3%)	1 (2%)	
WHO classifications			0.001
AML with t(8;21)	6 (7%)	3 (5%)	
AML with t(15;17)	27 (30%)	4 (7%)	
AML with minimal differentiation	1 (1%)	0 (0%)	
AML without maturation	7 (8%)	3 (5%)	
AML with maturation	25 (28%)	19 (35%)	
Acute myelomonocytic leukemia	14 (16%)	18 (33%)	
Acute monoblastic/monocytic leukemia	7 (8%)	7 (13%)	
Acute erythroid leukemia	3 (3%)	1 (2%)	
Karyotypic classifications			0.004
Favorable	33 (37%)	7 (13%)	
Intermediate	48 (53%)	35 (64%)	
Poor	7 (8%)	10 (18%)	
No data	2 (2%)	3 (5%)	
Karyotypes			0.009
normal	40 (44%)	27 (49%)	
t(8;21)	6 (7%)	3 (5%)	
t(15;17)	27 (30%)	4 (7%)	
complex	6 (7%)	9 (16%)	
others	9 (10%)	9 (16%)	
No data	2 (2%)	3 (5%)	
Gene mutations			
CEBPA^*^ (+/−)	13/68	4/47	0.194
*NPM1* (+/−)	9/72	6/45	1.000
*FLT3*-ITD (+/−)	7/74	10/41	0.107
*c-KIT* (+/−)	2/79	2/49	0.640
*N/K-RAS* (+/−)	6/75	4/47	1.000
*IDH1/2* (+/−)	3/78	6/45	0.088
*DNMT3A* (+/−)	5/76	6/45	0.335
*U2AF1* (+/−)	3/78	2/49	1.000
CR (+/−)	43/31 (58%)	14/38 (27%)	0.001

AML: acute myeloid leukemia; WBC: white blood cells; BM: bone marrow; FAB: French-American-British; WHO: World Health Organization; CR: complete remission. ^*^: single and double *CEBPA* mutations.

### Association of ID2 expression with genetic mutations

As was shown in Table 1, ten gene mutations (Table 1) were detected in 132 patients with available DNA. We did not observe the significant association of *ID2* expression with the tested gene mutations, besides that high *ID2* expression tended to be associated with *IDH1/2* and *FLT3*-ITD mutations (*P*=0.088 and 0.107, respectively).

### Correlation of ID2 expression with chemotherapy response

Follow-up data for complete remission (CR) rate after induction chemotherapy was obtained in 126 patients. *ID2*^high^ patients had significantly lower CR rate than *ID2*^low^ patients in whole-cohort AML (Table 1) and non-M3-AML [25% (12/36) vs 46% (25/29), *P*=0.039]. Moreover, for CN-AML, *ID2*^high^ patients tended to have lower CR rate than *ID2*^low^ patients [57% (20/15) vs 31% (8/18), *P*=0.068].

### Association of ID2 expression with clinical outcome

**Figure 3:**
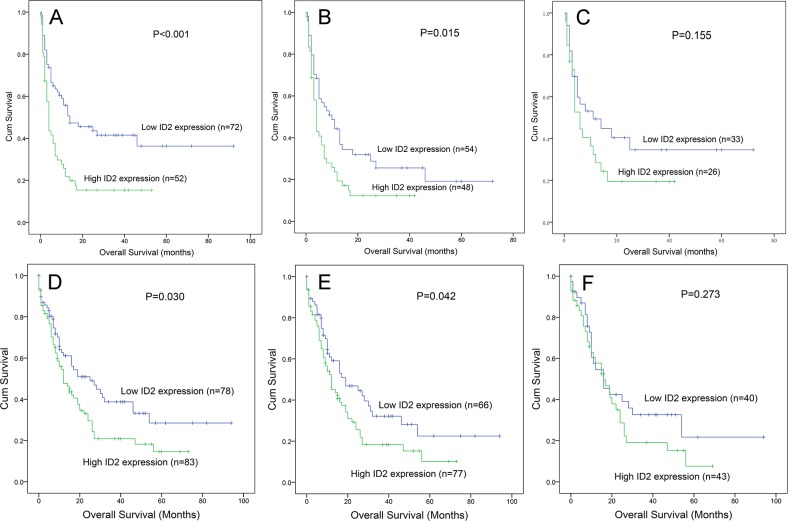
The impact of *ID2* expression on overall survival in AML patients **(A-C)** among our cohort. **(D-F)** data from TCGA databases. (A and D) whole-cohort AML patients; (B and E) non-M3-AML patients; (C and F) CN-AML patients.

Follow-up data for overall survival (OS) was obtained in 124 patients. Cases with high *ID2* expression had significantly shorter OS than those with low *ID2* expression among whole-cohort AML (*P*<0.001, Figure 3A) and non-M3-AML (*P*=0.015, Figure 3B). However, among CN-AML, although *ID2*^high^ patients showed shorter OS than *ID2*^low^ patients, the *P* did not attach statistical significance (*P*=0.155, Figure 3C).

We further performed Cox regression analyses (univariate and multivariate analyses) to determine the prognostic value of *ID2* expression in AML. The univariate analysis included the variables showed in Table 2, whereas the multivariate analysis included variables with *P*<0.100 in univariate analysis. After adjusting for other covariates, *ID2* expression acted as an independent prognostic biomarker in whole-cohort AML and non-M3-AML (Table 2) but not in CN-AML (data not shown).

**Table 2:** Univariate and multivariate analyses of prognostic factors for overall survival in AML patients

	Univariate analysis		Multivariate analysis	
	hazard ratio (95% CI)	*P* value	hazard ratio (95% CI)	*P* value
**Whole-cohort AML**				
*ID2* expression	2.133 (1.380-3.295)	0.001	1.889 (1.185-3.010)	0.008
Age	2.576 (1.650-4.020)	<0.001	1.754 (1.090-2.820)	0.020
WBC	2.375 (1.526-3.698)	<0.001	1.824 (1.148-2.899)	0.011
Karyotype classifications	2.220 (1.689-2.918)	<0.001	1.791 (1.316-2.437)	<0.001
*CEBPA* mutation^*^	0.891 (0.391-2.032)	0.784		
*NPM1* mutation	1.599 (0.822-3.108)	0.166		
*FLT3*-ITD mutation	0.891 (0.459-1.728)	0.732		
*C-KIT* mutation	1.155 (0.283-4.718)	0.841		
*DNMT3A* mutation	1.376 (0.662-2.860)	0.393		
*N/K-RAS* mutations	1.351 (0.587-3.112)	0.479		
*U2AF1* mutation	3.020 (1.200-7.598)	0.019	2.545 (0.972-6.660)	0.057
*IDH1/2* mutations	2.600 (1.239-5.456)	0.012	2.329 (1.033-5.254)	0.042
**Non-M3-AML**				
*ID2* expression	1.697 (1.082-2.663)	0.021	1.774 (1.101-2.859)	0.019
Age	1.940 (1.229-3.063)	0.004	1.589 (0.979-2.580)	0.061
WBC	1.787 (1.135-2.815)	0.012	1.720 (1.701-2.762)	0.025
Karyotype classifications	1.929 (1.394-2.669)	<0.001	1.774 (1.259-2.499)	0.001
*CEBPA* mutation^*^	0.730 (0.320-1.667)	0.455		
*NPM1* mutation	1.238 (0.633-2.418)	0.533		
*FLT3*-ITD mutation	1.077 (0.553-2.100)	0.827		
*C-KIT* mutation	0.945 (0.231-3.866)	0.937		
*DNMT3A* mutation	1.092 (0.524-2.276)	0.814		
*N/K-RAS* mutations	1.045 (0.451-2.418)	0.918		
*U2AF1* mutation	2.698 (1.069-6.810)	0.036	2.913 (1.111-7.636)	0.030
*IDH1/2* mutations	2.121 (1.006-4.474)	0.048	2.433 (1.072-5.524)	0.034

AML: acute myeloid leukemia; CI: confidence interval; WBC: white blood cells; ^*^: double *CEBPA* mutations. Variables including age (≤60 vs. >60 years), WBC (≥30×10^9^ vs. <30×10^9^ /L), *ID2* expression (lower vs. higher), and gene mutations (mutant vs. wild-type). Multivariate analysis includes variables with *P*<0.100 in univariate analysis.

### Validation of the prognostic value of ID2 expression by TCGA databases

To validate the prognostic value of *ID2* expression in AML, we searched and analyzed an independent assessment in AML patients from The Cancer Genome Atlas (TCGA) databases. By the median level of *ID2* expression set as the cut-off value, patients with higher *ID2* expression showed a significantly shorter OS among both whole-cohort AML (*P*=0.030, Figure 3D) and non-M3-AML (*P*=0.042, Figure 3E). Nevertheless, no significant difference was observed between the two groups for OS time among CN-AML (*P*=0.273, Figure 3F).

### ID2 expression in the surveillance of AML

**Figure 4:**
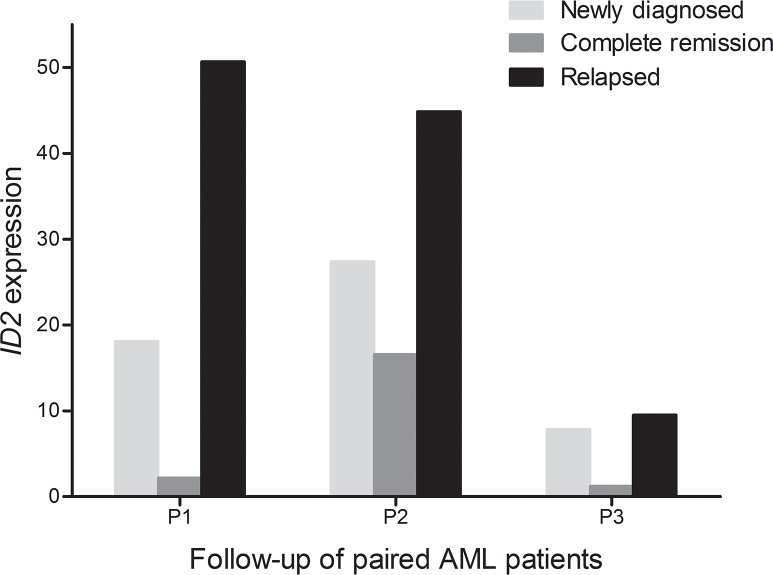
Dynamic change of *ID2* expression in the three paired AML patients of different stages (newly diagnosis, complete remission, and relapse time)

We next observed the dynamic change of *ID2* expression in the follow-up of three paired AML patients in different stages. Although the limited samples for detection, our data indicated that *ID2* expression at CR phase was decreased than at the time of diagnosis, and was increased at the time of relapse (Figure 4).

## DISCUSSION

*ID2* plays dual roles in different cancer types which may attribute to tissue-specific patterns of expression in different tissues and organs in tumourigenesis [4]. In lymphoid malignancies, although Nilsson et al. argued that *ID2* was dispensable for *MYC*-induced lymphomagenesis [8], a recent study reported that *ID2* and *ID3* proteins had a pro-survival function in chronic lymphocytic leukemia cells and also highlighted these proteins as potential determinants of the pathobiology of chronic lymphocytic leukemia [9]. In myeloid malignancies, *ID1* and *ID2* were lowly expressed in acute promyelocytic leukemia (APL) cells (NB4) and were rapidly induced upon all-trans retinoic acid (ATRA) treatment [10]. However, *ID2* was highly expressed in acute monocytic leukemia cells (THP-1) and was reduced by ATRA treatment [11]. Moreover, May et al reported that *ID2* and *ID3* proteins seemed to be involved in the granulopoietic maturation [12]. Similarly, our study also found that high *ID2* expression was less occurred in M3/t(15:17), and the low differentiated subtypes (M0/M1/M2) had a significantly higher frequency of high *ID2* expression than the highly differentiated subtype (M3).

In this study, we first identified that *ID2* overexpression was a frequent event in BMMNCs of *de novo* AML patients, and found it could act as a potential biomarker contributing to the diagnosis according to ROC curve analyses. When analyzed the clinical relevance of *ID2* expression in AML patients, we found the significant correlation of *ID2* overexpression with older age. The possible reason was that increasing age was associated with accumulation of aberrant gene expression. In addition, consistent with our previous study regarding *ID1* expression in the different risk groups of AML [5], we also observed the increased frequency of *ID2* overexpression with the rising risk of karyotype. Taken together, these results suggested that the role of *ID1* and *ID2* in the process of leukemogenesis may be dependent on the context of different cytogenetics.

Significantly, a negative effect of *ID2* overexpression on CR rate was observed among whole-cohort AML, non-M3-AML and CN-AML patients, which indicated that *ID2* overexpression was associated with poor induction chemotherapy response, and *ID2* expression might play a crucial role in affecting leukemia cell chemosensitivity in AML. However, the underlying mechanism of *ID2* expression mediated chemosensitivity in AML remains poorly investigated. A recent study reported that decreased *ID2* expression enhanced chemosensitivity to semustine, teniposide, and temozolomide in the U87 cell line by promoting apoptosis of glioblastoma cells [13]. Accordingly, further functional studies are needed to determine the underlying molecular mechanism of *ID2* in regulating chemosensitivity to cytarabine, daunorubicin, etc. in leukemia cells.

The impact of *ID2* expression on clinical outcome has been revealed in human cancers. Although Stighall et al disclosed that high *ID2* protein expression was associated with a favorable prognosis in primary breast cancer [14], a majority of investigations showed an adverse effect of *ID2* overexpression on clinical outcome in diverse cancer types. For instance, in breast cancer, another independent study reported both *ID1* and *ID2* overexpression were related to the advanced tumor stage, shorter OS and disease free survival [15]. In glioma, the increased *ID2* expression was closely associated with tumor grades, and correlated with shorter OS time [13]. Furthermore, Liu et al disclosed that overexpression of *ID2* was an unfavorable prognosis factor and promoted cell proliferation in nasopharyngeal carcinoma [16]. Our data also observed that high *ID2* expression was an independent prognostic biomarker in whole-cohort AML and non-M3-AML patients. Importantly, the results were further validated by TCGA data online available. Interestingly, recent studies showed *ID2* functioned as a negative regulator of leukemia stem cell potential in MLL-rearranged AML, and *ID2* overexpression suppressed MLL-rearranged and t(8;21) AML [17, 18]. Clinically, low expression of *ID2* or of an *ID2* gene signature was associated with poor prognosis in not only MLL-rearranged but also t(8;21) AML patients [17, 18]. However, in this study, we could not further analyze the impact of *ID2* expression on MLL-rearranged and t(8;21) AML due to the limited samples. Through the above results, we deduced that *ID2* might play different roles during leukemogenesis which may depend on cytogenetics. In addition, both our data and the TCGA dataset did not show the prognostic impact of *ID2* expression on OS among CN-AML patients, which may due the limited samples in CN-AML, or the specific association of *ID2* expression with cytogenetic abnormalities. In addition, our data showed that *ID2* expression might serve as a potential biomarker in disease surveillance in spite of the limited samples. Consequently, due to all the limitations, further studies are required to confirm our results before *ID2* could be a potential molecular target for gene therapy against leukemia.

In summary, *ID2* overexpression was a frequent event in AML patients, and was associated with higher karyotype risks. In spite of the association, high *ID2* expression predicts poor chemotherapy response and prognosis in *de novo* AML patients.

## MATERIALS AND METHODS

### Study population

A total of 145 AML patients and 33 healthy donors were enrolled in this study approved by the Institutional Review Board of the Affiliated People’s Hospital of Jiangsu University. The FAB and the 2008 revised WHO criteria were utilized for the diagnosis and classification of AML patients [19, 20]. After written informed consent obtained from all participants, BM was collected from all the healthy donors and patients at newly diagnosis time, as well as three patients at time of CR and relapse. BM mononuclear cells (BMMNCs) obtained from participants were separated by Ficoll solution and washed twice with PBS.

### Treatment

All the patients received chemotherapy as reported in our previous literature [7, 21]. For APL patients, induction therapy was oral ATRA together with daunorubicin in combination with cytarabine, and maintenance therapy was oral mercaptopurine, oral methotrexate, and oral ATRA over two years. For non-APL patients, induction therapy was one or two courses of daunorubicin combined with cytarabine, whereas subsequent consolidation treatment included high-dose cytarabine, mitoxantrone combined with cytarabine, homoharringtonine together with cytarabine, and etoposide in combination with cytarabine.

### Cytogenetic and mutation analyses

Karyotypes were analyzed by conventional R-banding method and karyotype risk was classified according to reported previously [22]. Mutations in *NPM1*, *C-KIT*, *DNMT3A*, *IDH1/2*, *N/K-RAS* and *U2AF1* were detected by high-resolution melting analysis as reported [23–29], whereas *FLT3*-ITD and *CEBPA* mutations were examined by DNA sequencing [30, 31].

### RNA isolation and reverse transcription

Total RNA was isolated from the BMMNCs using Trizol reagent (Invitrogen, Carlsbad, CA, USA). cDNA was synthesized by reverse transcription using random hexamers as reported [32].

### RQ-PCR

RQ-PCR was performed to detect *ID2* and *ABL1* mRNA level. The primer of *ID2* expression were 5′-CTGGACTCGCATCCCACTAT-3′ (forward) and 5′-CACACAGTGCTTTGCTGTCA-3′ (reverse) as reported [33]. PCR reaction was consisted of cDNA 20 ng, 0.4 μM of primers, 10 μL of AceQ qPCR SYBR Green Master Mix together with 0.4 μL of ROX Reference Dye 2 (Vazyme Biotech Co., Piscataway, NJ, USA). PCR conditions were conducted at 95°C for 5 min, followed by 40 cycles at 95°C for 10 s, 65°C for 30 s, 72°C for 32 s, and 83°C for 32 s. Housekeeping gene *ABL1* detected as reported [34] was used to calculate the abundance of *ID2* mRNA. Both positive [K562 cell lines samples, cultured in RPMI 1640 medium containing 10% fetal calf serum (ExCell Bio, Shanghai, China)] and negative controls (ddH_2_O) were included in each assay. Relative *ID2* expression levels were calculated using 2^−ΔΔCT^ method.

### TCGA databases

*ID2* mRNA expression (RNA Seq V2 RSEM) in a cohort of 200 AML patients [35] from TCGA databases were obtained via online website cBioPortal (http://www.cbioportal.org) [36, 37].

### Statistical analyses

Statistical analyses were performed through SPSS 20.0 software package. Mann-Whitney’s *U* test and Pearson Chi-square/Fisher exact test was employed to compare the difference of continuous and categorical variables between two groups. ROC and AUC were conducted to assess the value of *ID2* expression in distinguishing whole-cohort AML, non-M3-AML and CN-AML patients from controls. Kaplan-Meier and Cox regression analyses were performed to analyze the impact of *ID2* expression on OS. For all analyses, a two-tailed *P* value less than 0.05 was determined as statistically significant.

## Abbreviations

AML: acute myeloid leukemia
CR: complete remission
TCGA: The Cancer Genome Atlas
ID: Inhibitor of DNA binding
BM: bone marrow
RQ-PCR: real-time quantitative PCR
CN-AML: cytogenetically normal AML
ROC: receiver operating characteristic curve
AUC: an area under the ROC curve
CI: confidence interval
FAB: French-American-British
WHO: World Health Organization
OS: overall survival
APL: acute promyelocytic leukemia
ATRA: all-trans retinoic acid
BMMNCs: BM mononuclear cells.
